# Eye-Tracking Provides a Sensitive Measure of Exploration Deficits After Acute Right MCA Stroke

**DOI:** 10.3389/fneur.2018.00359

**Published:** 2018-06-11

**Authors:** Margarete Delazer, Martin Sojer, Philipp Ellmerer, Christian Boehme, Thomas Benke

**Affiliations:** Department of Neurology, Medical University Innsbruck, Innsbruck, Austria

**Keywords:** acute MCA stroke, visual exploration, neglect, eye-tracking, cancellation task, assessment

## Abstract

The eye-tracking study aimed at assessing spatial biases in visual exploration in patients after acute right MCA (middle cerebral artery) stroke. Patients affected by unilateral neglect show less functional recovery and experience severe difficulties in everyday life. Thus, accurate diagnosis is essential, and specific treatment is required. Early assessment is of high importance as rehabilitative interventions are more effective when applied soon after stroke. Previous research has shown that deficits may be overlooked when classical paper-and-pencil tasks are used for diagnosis. Conversely, eye-tracking allows direct monitoring of visual exploration patterns. We hypothesized that the analysis of eye-tracking provides more sensitive measures for spatial exploration deficits after right middle cerebral artery stroke. Twenty-two patients with right MCA stroke (median 5 days after stroke) and 28 healthy controls were included. Lesions were confirmed by MRI/CCT. Groups performed comparably in the Mini–Mental State Examination (patients and controls median 29) and in a screening of executive functions. Eleven patients scored at ceiling in neglect screening tasks, 11 showed minimal to severe signs of unilateral visual neglect. An overlap plot based on MRI and CCT imaging showed lesions in the temporo–parieto–frontal cortex, basal ganglia, and adjacent white matter tracts. Visual exploration was evaluated in two eye-tracking tasks, one assessing free visual exploration of photographs, the other visual search using symbols and letters. An index of fixation asymmetries proved to be a sensitive measure of spatial exploration deficits. Both patient groups showed a marked exploration bias to the right when looking at complex photographs. A single case analysis confirmed that also most of those patients who showed no neglect in screening tasks performed outside the range of controls in free exploration. The analysis of patients’ scoring at ceiling in neglect screening tasks is of special interest, as possible deficits may be overlooked and thus remain untreated. Our findings are in line with other studies suggesting considerable limitations of laboratory screening procedures to fully appreciate the occurrence of neglect symptoms. Future investigations are needed to explore the predictive value of the eye-tracking index and its validity in everyday situations.

## Introduction

Neglect has been defined as the inability to report, respond, or orient to novel or meaningful stimuli presented to the side opposite a brain lesion (1, 2). Patients with spatial neglect after lesions of the right hemisphere show a pronounced bias of spontaneous exploratory activity toward the right, ipsilesional side of space. Neglect is more often observed after right than after left hemispheric lesions. Critical regions include the inferior parietal lobule and temporoparietal junction (3, 4), the superior temporal gyrus and adjacent insular cortex (5), and the inferior frontal gyrus (6). In addition, damage to subcortical nuclei and white matter disruption between frontal, temporal, and parietal cortex may lead to neglect symptoms (7–9). Patients affected by neglect experience severe difficulties in everyday life, show less functional recovery and poor social adjustment (10–12). Thus, accurate and early diagnosis is essential and specific treatment is required (13).

The present study aims at assessing spatial biases in visual exploration in patients after acute right MCA (middle cerebral artery) stroke. Visual exploration biases are investigated in eye-tracking tasks as well as in paper-and-pencil tasks. The study focusses on acute stroke patients. Early assessment is of high importance as rehabilitative interventions are more effective when applied soon after stroke (14, 15).

A hallmark of unilateral visual neglect is the inability to pay attention to targets contralateral to the brain lesion [for reviews see Ref. (16, 17)]. A number of neuropsychological scales, screenings and tests have been proposed, among them cancelation tasks, line bisection tasks, drawing, copying, computerized target detection, or reading tasks (18–23). These tasks have been found to have a substantially varying sensitivity in detecting spatial neglect. Several task-related factors have an impact on patients’ performance, among them the task demands [challenging tasks having a higher sensitivity than easy tasks (20, 24)], the task instructions [the behavioral goal modulates the deficits (25, 26)], visual feedback (27), and the specific features of the stimulus material [stimulus density, dynamics, brightness, and contrasts influence the performance (28, 29)]. Both, top-down (concept-driven encoding of stimuli based on context, beliefs, knowledge, or desires) and bottom-up processes (modulated by structural features of the stimuli) are found to modulate behavior (30). Given the heterogeneity of the syndrome and the multitude of possibly influencing factors, the lack of consensus as regards appropriate testing methods (18) is not surprising. Highly relevant for clinicians, neglect can be dramatically overlooked when based only on standard paper-and-pencil testing where patients can easily compensate for their deficits (24).

The use of eye-tracking tasks has proven to be a fruitful approach in research on neglect phenomena in the past [e.g., (30–34)]. Eye-tracking allows direct monitoring of visual exploration and thus reflects the distribution of attention over space. Eye-tracking studies have used various types of stimulus material as random arrays of letters, geometrical shapes, everyday photographs (30) or movies (28), various fields of exploration, and various paradigms. Karnath (17) has shown that in free visual exploration neglect patients shift their attention by approximately 15° to the ipsilesional side. Moreover, visual exploration in neglect is not only biased toward the ipsilesional side but is also characterized by frequent refixations, i.e., recurrent returns to already explored areas (35, 36), by shorter saccade amplitudes and by longer fixation durations. While eye-tracking studies have provided major insight and progress in neurocognitive research, the use of eye-tracking tasks for clinical purposes has gained less interest.

Based on previous observations, we assumed that patients in the acute stage after right MCA stroke show a bias to the right space in visual exploration tasks. We expected that, due to its accuracy, eye-tracking may provide a more sensitive measure of alterations in spatial exploration than classical neglect screening tasks. The analysis of patients’ performance without obvious neglect on standard clinical tasks is of special interest, as possible deficits may be overlooked and thus remain untreated. We analyzed visual exploration in two eye-tracking tasks, one assessing free visual exploration, the other assessing visual search. We hypothesized that an index of fixation asymmetries has a discriminative power and reliably separates between patients after stroke and healthy participants. We tested this hypothesis for patients with minimal to severe signs of neglect and separately for those who show no asymmetry in neglect screening tasks.

## Materials and Methods

### Participants

We included 22 consecutively admitted patients with an acute, first right sided MCA infarct. Imaging was performed in the acute phase by standard MRI or CCT. Patients with additional lesions or with diffuse brain lesions were excluded from the investigation. The patients were assessed as soon as they could collaborate in the study. They were clinically stable, could leave the stroke unit, had sufficient vigilance and attention, and were physically able to sit relatively stable in front of the computer screen. Table 1 gives the time since stroke, the National Institutes of Health Stroke Scale scores (NIHSS), and the modified Rankin Scale scores (mRS) at the time of admission and of discharge. Visual field defects were assessed clinically by confrontation testing; 20 patients showed no visual field defect, 2 patients had left hemianopia. Patients with hemianopia were excluded in a separate analysis. All patients were right-handed. A group of 28 neurologically healthy controls matched in age and gender was assessed (see Table 1). All participants gave informed consent to participate in the study. The study was approved by the ethics committee of the Medical University Innsbruck.

**Table 1:** Clinical data and background tasks.

	NN-patients (*n* = 11; 6 male)			NE-patients (*n* = 11; 6 male)				Controls (*n* = 28; 11 male)		
	Median	Quartile range		Median	Quartile range		M.W. NN vs. NE; *p*			
**Clinical data**										
NIHSS admission	1.00	0.00–7.00		9.00	2.00–13.00		0.040			
mRS admission	2.00	2.00–4.00		3.00	2.00–5.00		0.171			
NIHSS discharge	0.00	0.00–1.00		2.00	1.00–5.00		0.019			
mRS discharge	1.00	0.00–2.00		2.00	1.00–2.00		0.193			
Days since stroke (days)	4.00	3.00–6.00		6.00	3.00–13.00		0.243			

	**Median**	**Quartile range**	**M.W. NN** vs. **C; ***p*****	**Median**	**Quartile range**	**M.W. NE** vs. **C; ***p*****	**M.W. NN** vs. **NE; ***p*****	**Median**	**Quartile range**	**K.W. ***p*****

**Background tasks**										
Age	68.00	47.00–77.00	–	62.00	53.00–78.00	–	–	55.00	52.50–70.50	0.559
MMSE (max. 30)	29.00	29.00–30.00	*–*	29.00	26.00–30.00	*–*	–	29.00	29.00–30.00	0.424
Clock drawing (Clox, max. 15)	12.00	11.00–14.00	0.033	11.00	8.00–12.00	<0.0001	0.088	14.00	13.00–14.00	<0.0001
Frontal assessment B. (FAB, max. 18)	17.00	16.00–18.00	–	16.50	13.00–18.00	–	–	18.00	17.00–18.00	0.103
Ota circle task; CoC	0.00	0.00–0.00	0.701	0.04	0.00–0.17	0.009	0.056	0.00	0.00–0.00	0.003
Defect detection task; A value	0.00	0.00–0.00	–	0.00	−0.01–0.04	–	–	0.00	0.00–0.00	0.726
Symbol cancelation; CoC	−0.01	−0.03–0.00	0.132	0.09	0.00–0.44	0.006	0.004	0.00	−0.03–0.02	0.004
Line crossing task; CoC	0.00	0.00–0.00	0.747	0.00	0.00–0.16	0.018	0.076	0.00	0.00–0.00	0.004
Reading numerals (max. 9)	9.00	9.00–9.00	*–*	9.00	9.00–9.00	*–*	–	9.00	9.00–9.00	*–*

*Age, National Institutes of Health Stroke Scale Scores (NIHSS) and modified Rankin Scale scores (mRS) at the time of admission and at the time of discharge, time since stroke for patient groups (NN, no neglect; NE, minimal to severe neglect). Background tasks: performance (median, quartile ranges) of patients and controls in background tasks and neglect screening tasks. Group comparisons (age, background tasks) were performed by Kruskal–Wallis Tests (K.W.; NN-patients, NE-patients, controls) followed by Mann–Whitney Tests between controls and patient groups (NN-patients vs. controls and NE-patients vs. controls) and between the two patient groups (NN- vs. NE-patients). CoC values indicate the Center of Cancelation [Rorden and Karnath (22)]. The CoC value accounts for both the number of errors in cancelation tasks and the spatial distribution of the errors. Uncorrected *p*-values. MMSE, Mini–Mental State Examination*.

### Background Tasks

Patients and controls performed the Mini–Mental State Examination (MMSE; controls only above the age of 60), the Frontal Assessment Battery [FAB; (37, 38)] and a clock drawing task [CLOX; (39)]. Scores (median and quartile range) for the three tasks are shown in Table 1.

### Neglect Screening

A test battery including four paper-and-pencil cancelation tasks [Symbol Cancelation, Line Crossing, Ota Circle Task and Defect Detection Task (22, 40–42)], the clock drawing task, the copying task of the MMSE, and a number reading task (three pages with three horizontally presented two-digit numbers presented on the computer screen) was used for neglect screening. All paper-and-pencil tasks were printed on a horizontally oriented 21 cm × 29.7 cm sheet of paper and were presented centrally in front of the participant. No time limit was given for the screening tasks. Tasks were administered until the patient confirmed completion of the task. Performance in the cancelation tasks was evaluated by calculating the CoC (Center of Cancelation) score, a continuous measure of neglect severity [(22)].^1^ The CoC value accounts for both the number of errors in cancelation tasks and the spatial distribution of these errors. For perfectly symmetrical tests, a patient who detects only the leftmost target gets a score of minus one, a patient who detects the rightmost item gets a score of one. Participants who miss no items or have a perfectly symmetrical pattern of omissions have a score near 0. For scoring the Defect Detection Task, we used the same software. The software provides a value indicating allocentric neglect [A-value; (43)]. Based on previous studies and the data of healthy controls we applied the following procedure: Patients were assigned one point each if they had a CoC value >0.02 in the Line Crossing Task, a CoC value >0.01 in the Ota Circle Task, a CoC value >0.07 in the Symbol Cancelation Test or an A-value >0.055 in the Defect Detection Task [highest scores of controls were used as lower cutoffs; Rorden and Karnath (22) reported higher cutoffs for CoC values in two cancelation tasks]. They were further assigned one point each if they showed left-sided omissions in clock drawing (regardless of the overall CLOX score), if they omitted one or more left-sided numbers in the number reading task (whole number or single digits) or if they had left-sided omissions in the copying task of the MMSE (regardless of the overall MMSE score). The total maximum score of the neglect screening was 7. Patients were classified as showing no signs of neglect if they scored 0 (No Neglect; NN-patients), as showing signs of neglect when they scored 1 or higher in the sum of the screening tasks (Neglect; NE-patients). Note that this classification is rather strict and that patients showing minimal signs of neglect fall into the NE-group. Neglect screening tasks and eye-tracking tasks were performed in the same session.

### Eye-Tracking

Eye movements were recorded with a TobiiTX300 remote eye-tracker with a sampling rate of 300 Hz with a screen resolution of 1,920 × 1,080 pixels. Participants were positioned comfortably approx. 65 cm from the screen and could rest their head on the backrest. As the system is provided with a head movement compensation mechanism, sufficient reliability in data collection was achieved (44). A 9-point calibration was performed. The quality of calibration was visually checked and repeated if necessary. The median proportion of successfully recorded data was 90% in the search task (quartile range 83–94) and 91% in the free exploration task (quartile range 81–94). Participants’ performance was constantly monitored with real-time viewing on a second screen, with the stimuli and the gaze data superimposed. Most patients were also video recorded and performance was checked with the remote live viewer.

### Eye-Tracking Tasks

#### Free Exploration Task

10 pictures of landscapes or complex scenes (e.g., furniture, flea market, landscape with sheep) were presented consecutively on the screen (pictures were downloaded from https://pixabay.com/de/; california-106943; cattle-show-1715039; computer-627220; drugs-1679815; flea-market-851978; flea-market-1681489; holz-659495; huskies-273409; shopping-1232944; wash-1141774). Total visit duration did not differ between left and right hemifield in a pilot study with 20 healthy controls. The visual angle was 28.3°, horizontal extension 34 cm (1,280 × approx. 855 pixels). Presentation time was 10 s and participants were asked to report what they noticed on the pictures. Participants were not forced to report as many details as possible.

#### Visual Search Task

Six trials were presented in the visual search task (position and visual angle as in the free exploration task; 1,280 × 720 pixels; presentation time 15 s). Participants were asked to search for the respective targets (symbols or letters) among distractors and name their number when found (the number of targets was between 0 (no target present in 2 trials) and 9; the number of distractors was between 23 and 34).

### Lesion Analysis

Brain MRI scans included T1, T2, fluid attenuated inversion recovery, and diffusion images obtained with standard parameters on a 1.5 T Siemens Magnetom Symphony Tim scanner. Lesion extent was determined for each patient by selecting brain scans that showed the greatest extent of damage and drawing the lesion borders directly onto the original 3D images, using the MRIcro software (45) available on-line.^2^ Five patients could not undergo MRI and had only a brain CT scan available. Their lesions were delineated using a similar procedure, first drawn from the CT image and then transposed to the standard MRI template of MRIcro. All lesion maps were drawn and double-checked by two neurologists (Thomas Benke, Martin Sojer) trained to read brain scans. The 3D brain scan and lesion volume were then normalized to a standard brain template using a combination of MRIcro and Statistical Parametric Mapping-2^3^ running under Matlab.^4^ The normalized lesion images were used as a region of interest for subsequent analysis in MRIcro. The lesions, drawn as regions of interest for each patient, were then displayed on a common template in order to determine areas of lesion overlap.

### Statistics and Data Analysis

A Velocity Threshold Identification Fixation (IVT) filter was applied to the eye-tracking data.^5^ Data points with angular velocity below 30°/s were classified as fixation and data points above were classified as saccade. The minimum fixation duration was set to 60 ms. Data from both eyes were averaged. Fixation duration, number of fixations, and saccadic amplitude (calculated as the distance between adjacent fixations in pixels) were measured. Furthermore, we computed two spatial exploration indices reflecting spatial biases. Indices describe the relation between the number of fixations in the left area of interest (L_AOI, leftmost 400 pixels of the stimulus) and in the right area of interest (R_AOI, rightmost 400 pixels of the stimulus). The index was computed separately for the two tasks: (number of fixations in R_AOI − number of fixations L_AOI)/(number of fixations in R_AOI + number of fixations L_AOI). A value of +1 indicates exclusive exploration in the right area of interest, a value of 0 a completely symmetrical pattern of visual exploration, and a value of −1 exclusive exploration in the left area of interest. The index (Index-Free in the free exploration task; Index-Search in the search task) is a more reliable measure than the raw numbers of fixations as possibly confounding variables including absolute number of fixations, overall fixation duration or scanning velocity are controlled.

Since most measures were not normally distributed (tested with Shapiro–Wilk tests), we ran non-parametric analyses, using Kruskal–Wallis tests (NE-patients, NN-patients, controls) followed by Mann–Whitney tests between groups when significant results were found. Within groups analyses were performed by Wilcoxon tests. For the NE-patients we performed a Spearman’s rank-order correlation analysis between spatial exploration indices and performance in the neglect screening tasks. A further analysis assessed the correlation between NIHSS admission score and spatial indices (for the two patient groups separately). A single case analysis evaluated the distribution of the spatial exploration indices in patients and controls. Receiver operating characteristics (ROC) curve analyses were used to evaluate the discriminating power of the spatial exploration indices separately for the NN-patients and the NE-patients. The area under the curve (AUC) was used to measure the overall performance of each ROC curve. AUC values were classified as excellent (0.90–1), good (0.80–0.90), fair (0.70–0.80), or poor (below 0.70).

## Results

### Lesion Analysis

**Figure 1:**
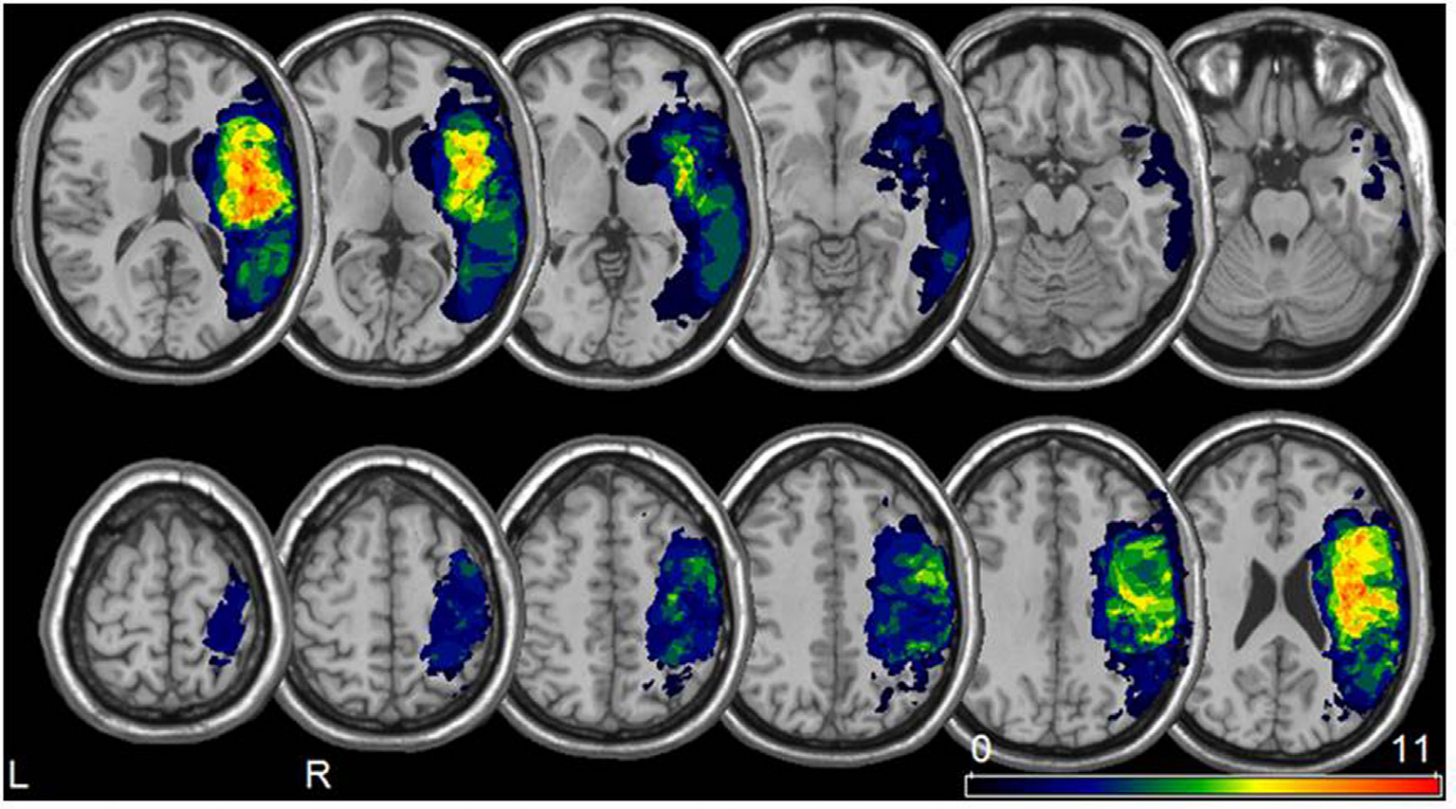
Lesion analysis. Lesion overlay map for all 22 patients included in our study (neurological convention). The color bar represents the degree of lesion overlap.

The greatest lesion overlap was found in the fronto–parieto–temporal cortex, insula and the basal ganglia. White matter lesions mainly included the superior longitudinal and inferior fronto-occipital fasciculus, the internal capsule/corona radiata, and the pyramidal tract (Figure 1).

### Patient Classification

Patients were classified as showing no neglect (NN-patients; *n* = 11; median = 0) when they had a score of 0 in the screening tasks, and as showing minimal to severe signs of neglect when they scored 1 or higher in the screening tasks (NE-patients; *n* = 11; median 2, range 1 to 5). For controls the summary score was not computed as only those aged above 60 performed the MMSE.

### Comparisons Between Groups—Background Tasks and Neglect Screening Tasks

NN-patients performed lower than controls in the Clox task [scoring as in Ref. (39)]; NE-patients performed lower than controls in the Clox task, and in three neglect screening tasks (for median scores, quartile ranges and *p*-values, see Table 1).

### Eye-Tracking

**Figure 2:**
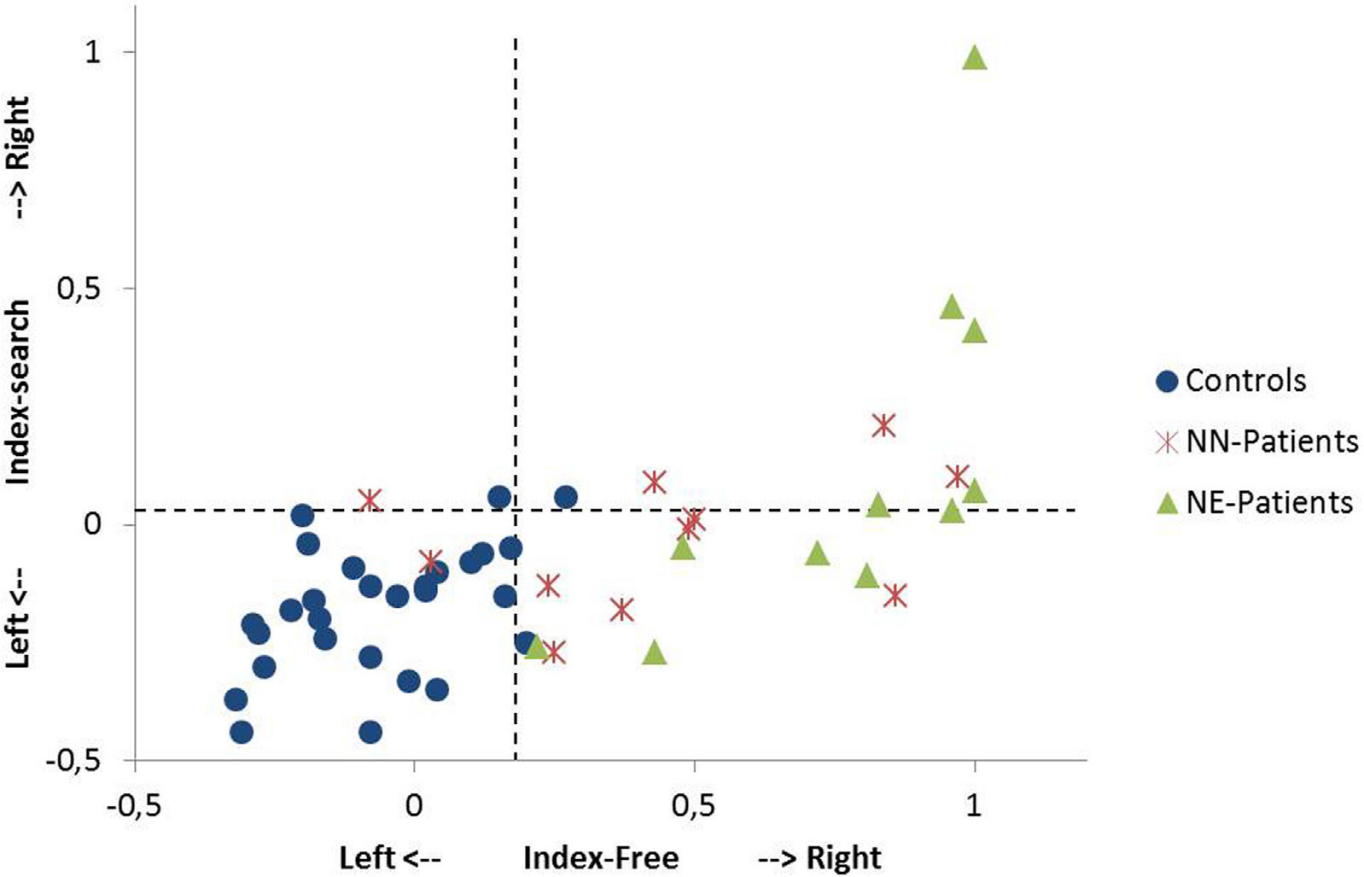
Single case analysis in the free exploration task and in the search task. The Index-Free (*x*-axis) describes the spatial preference in free visual exploration in horizontal space. The Index-Search (*y*-axis) describes the spatial preference in the search task in horizontal space. Positive values describe exploration to the right, negative values exploration to the left (for the computation of the indices, see Materials and Methods). A value of +1 indicates that the participant exclusively fixates in the right sided area of interest (400 pixels on the right side of the stimulus). The performance of single patients [NN-patients (no neglect); NE-patients (patients with minimal to severe neglect)] and controls are shown. Dotted lines indicate the 95th percentile of healthy controls in the free exploration task and in the search task, respectively.

NN-patients had longer fixation durations than controls in both eye-tracking tasks and a lower number of fixations in the search task. NE-patients showed longer fixation durations, lower numbers of fixations, and overall shorter saccade amplitudes than controls (both eye-tracking tasks; for median scores, quartile ranges and *p*-values, see Table 2).^6^ They also answered fewer items correctly in the search task. The spatial bias to the right was more pronounced in both patient groups than in controls in the visual exploration (Index-Free) and in the search task (Index-Search; Table 2; Figure 2). A comparison between patient groups did not show a significant difference in the search task (Index-Search), and approached significance in visual exploration task (Index-Free; Table 2).

**Table 2:** Eye-tracking measures patient groups and controls.

	NN-patients (*n* = 11; 6 male)			NE-patients (*n* = 12; 6 male)				Controls (*n* = 28; 11 male)		
	Median	Quartile range	*M.W*. *NN* vs. *Cp*	Median	Quartile range	*M.W. NE* vs. *C p*	*M.W. NE* vs. *NN p*	Median	Quartile range	*K.W. p*
**Eye-tracking free exploration**										
Fixation duration (ms)	276.25	263.70–335.29	0.010	332.86	258.25–472.36	0.002	0.171	234.86	215.08–267.99	0.002
Number of fixations (*n*; 10 s)	32.10	27.50–33.30	0.301	27.00	19.30–28.40	<0.0001	0.010	33.40	29.80–35.35	0.002
Saccadic amplitude (pixels)	229.78	201.07–263.15	–	177.68	165.29–233.40	0.022	–	224.68	199.90–262.64	0.076
Saccadic amplitude to the right	224.10	205.74–249.37	0.548	176.86	143.75–232.56	0.003	0.065	228.85	204.78–278.34	0.013
Saccadic amplitude to the left	221.15	193.70–276.77	–	203.31	182.78–244.31	–	–	229.27	188.20–250.06	0.644
**Eye-tracking visual search**										
Fixation duration (ms)	236.34	222.23–295.96	0.003	236.78	220.55–311.10	0.003	1	200.39	180.63–210.98	0.001
Number of fixations (*n*; 15 s)	46.17	40.83–52.67	0.001	48.00	39.33–52.61	0.001	1	58.25	52.00–60.75	<0.0001
Saccadic amplitude (pixels)	228.16	193.32–244.99	0.866	189.14	152.39–240.81	0.007	0.065	228.57	203.53–247.54	0.028
Saccadic amplitude to the right	233.52	203.53–255.92	–	190.08	156.19–250.55	–	–	225.73	207.45–247.77	0.149
Saccadic amplitude to the left	225.23	171.71–259.45	0.842	180.00	152.85–225.08	0.002	0.088	234.34	206.63–253.16	0.015
Items correct (max. 6)	5.00	5.00–6.00	0.914	4.00	4.00–5.00	0.001	0.008	5.00	5.00–6.00	0.002
**Eye-tracking free exploration**										
**Spatial indices**										
Index-Free	0.43	0.24–0.84	<0.0001	0.83	0.48–1.00	<0.0001	0.056	−0.08	−0.19–0.07	<0.0001
Index-Search	−0.01	−0.15–0.09	0.010	0.03	−0.11–0.41	0.005	0.519	−0.15	−0.27 to −0.09	0.004

*Performance (median, quartile ranges) of patients (NN, no neglect; NE, minimal to severe neglect) and controls in eye-tracking tasks. Group comparisons were performed by Kruskal–Wallis Tests (K.W.; NN-patients, NE-patients, controls) followed by Mann–Whitney Tests between patient groups and controls when significant results were found in K.W. tests (NN-patients vs. controls, NE-patients vs. controls, NN- vs. NE-patients). Uncorrected *p*-values*.

### Comparisons Between Free Exploration and Search Task

The exploration bias toward the right (as indicated by spatial indices) was stronger in free visual exploration than in visual search in all groups, NE-patients (Wilcoxon test; *p* = 0.003), NN-patients (*p* = 0.006), and controls (*p* = 0.001). The mean fixation duration was shorter in visual search than in free exploration in controls (*p* < 0.0001) and NE-patients (*p* = 0.004), but not in NN-patients (*p* = 0.062). Saccadic amplitude did not differ between tasks in any group (all *p* > 0.65).

### Single Case Analysis

9 out of 11 NN-patients showed a pronounced bias to the right in free exploration [Index-Free higher than the 95th percentile of controls (Index-Free = 0.18); see Figure 2] and 4 out of 11 in the search task [Index-Search higher than the 95th percentile of controls (Index-Search = 0.03)]. In the NE-group, all patients had a higher Index-Free, and 5 out of 11 had a higher Index-Search than the 95th percentile of controls (see Figure 2).

### ROC Curve

Receiver operating characteristics curve analyses were performed to test the discriminating power of the spatial exploration indices between control and patient groups. For the NN-patients, free exploration (Index-Free) had an AUC value of 0.92 (=excellent), the search task (Index-Search) an AUC of 0.76 (=fair). For the NE-patients, the AUC was >0.99 (=excellent) in the free exploration task and 0.79 (=fair) in the visual search task.

### Correlation Analysis, Role of Neglect, and Stroke Severity

For the NE-patients, we performed a Spearman’s rank-order correlation analysis between performance in the neglect screening tasks (summary score) and spatial indices. NN patients were excluded from the analysis as they had a neglect summary score of 0. For NE-patients, performance in the neglect screening tasks correlated with the spatial bias in the search task (Index-Search; Spearman; *r* = 0.66; *p* = 0.027) and in the free exploration task (Index-Free; Spearman; *r* = 0.60; *p* = 0.049). In a further analysis, we assessed the Spearman’s rank-order correlation between NIHSS admission scores and spatial indices. NN-patients showed a significant correlation in the free exploration task, but not in the search task (Index-Free *r* = 0.651, *p* = 0.030; Index-Search *r* = 0.130, *p* = 0.703), NE-patients showed significant correlations in both tasks (Index-Free *r* = 0.745, *p* = 0.009; Index-Search *r* = 0.597, *p* = 0.053).

### The Role of Hemianopia

Main results of the investigation did not change when excluding the two patients with hemianopia (one patient in each group). For both patient groups, Index-Free and Index-Search differed significantly from controls (NE-patients: Index-Free *p* < 0.0001, Index-Search *p* = 0.013; NN patients: Index-Free *p* < 0.0001, Index-Search *p* = 0.019; Mann–Whitney tests).

## Discussion

The present study investigated visual exploration in right MCA stroke patients in paper-and-pencil screening tasks and in two eye-tracking tasks. We included patients in the acute phase of a first ever unilateral right hemisphere stroke who suffered a single lesion in the right MCA territory. Patients with ACP infarcts were excluded. A lesion analysis confirmed that lesions were comparable to other neglect studies including cohorts with right MCA stroke (3, 5, 9, 46). At the group level both, NE- and NN-patients, presented a pronounced fixation asymmetry to the right in the free visual exploration of photographs. Visual exploration of complex scenes requires top-down (concept-driven encoding) as well as bottom-up processes (detection of relevant stimuli) which both may influence the outcome. Both, NE and NN group, also showed more right sided exploration than controls in a visual search task, though the spatial bias to the right was significantly less pronounced in the search task than in the free exploration task. The two tasks cannot be easily compared as task instructions (search vs. free exploration) and stimulus material (letters and symbols vs. complex photographs) were different. Possibly, letters and symbols in the search task induced a more systematic scanning extending to the left AOI. The significant correlation between performance in the eye-tracking search task and scores in the screening tasks (with letters and symbols) in the NE-group indeed suggests that similar processes were assessed. In free exploration, the spatial bias to the right was related to the severity of stroke as rated in the NIHSS admission score in both patient groups.

Receiver operating characteristics curve analyses were performed to test the discriminating power of the spatial exploration indices between control and patient groups. While the excellent discriminative power could be expected for identifying NE-patients, the high value for NN-patients was less expected and confirms the close association between acute right MCA stroke and spatial exploration deficits even when no neglect is obvious in screening tasks.

The single case analysis gives a more differentiated picture than the group analysis. Several patients had a strong bias to the right in free visual exploration, but neither (or less) in the visual search task, nor in the paper-and-pencil cancelation tasks. Thus, eye-tracking of exploring photographs seems to be a more sensitive approach to identify spatial attention asymmetries in single patients than using search/cancelation tasks. This result is in line with previous findings indicating that the automatic rightward orientation bias is the most sensitive clinical measure of neglect (18). Using eye-tracking measures instead of behavioral observation allows to objectively quantify the rightward bias and to exactly monitor the deficit. Eye-tracking is in most cases easy to perform also in the acute stage of the disease (here 5 days after stroke), does not require demanding and tiring testing procedures and allows exact quantification of exploration biases.

In the present investigation, we computed indices of fixation asymmetries as outcome measures. We put the number of fixations in the rightmost third (extending 14–15° to the right) in relation to the number of fixations in the leftmost third and to the overall sum of fixations. We thus assess the symmetry of fixation patterns and not only the peak of the distribution. The indices are more reliable measures than the raw numbers of fixations in specific AOIs which may differ between groups due to several reasons. In the present investigation both groups, NE- and NN-patients showed longer fixation durations in both tasks which may be a confounding factor when comparing the raw numbers of fixations.

Results indicate that NE-patients had smaller saccade amplitudes than controls in both tasks. Smaller saccade amplitudes in NE-patients in all directions have been reported previously (47). Conversely, several studies using target detection tasks have reported lateralized deficits in performing reflexive eye movements toward targets suddenly appearing in the left hemifield in neglect patients (48, 49). Different mechanisms are assumed to support voluntary and stimulus-driven saccades (50). Reflexive saccades may be impaired while saccades to the same spatial locations may be intact in the same patients in deliberate search (50). Thus, task demands seem to play an essential role.

NE-patients showed shorter saccades to the right than controls in the free exploration task. This fact is not surprising as NE-patients predominantly fixated spatial locations on the right side of the stimulus. Since the spatial extent of the stimulus is limited, unexplored stimulus area extended far more to the left than to the right. The situation was different in the search task, where NE-patients showed shorter saccades to the left than controls. In this task, their fixations were located more centrally. Shorter saccades to the left may reflect a neglect-associated symptom. Whether this is the consequence of deficient visual exploration mechanisms or causally contributes to altered fixation patterns remains an open question.

The present findings are of importance, as they signal the necessity to carefully assess right MCA stroke patients as soon as possible in order to initiate appropriate rehabilitation programs. Previous investigations have shown that computerized tasks where suddenly appearing stimuli have to be detected are highly sensitive screening instruments (20). The present study indicates that patients show also deficits in the voluntary exploration of a visual setting. This may be particularly relevant for seemingly intact, autonomous patients performing well in standard tasks. In real life, detection of suddenly appearing objects is important, but also the careful voluntary exploration of the entire scene. As suggested by our results, patients after right MCA stroke may show severe difficulties in the latter capacity though performing well in neglect screening tasks.

The analysis of eye-tracking tasks proved to be highly informative about the characteristics of visual exploration patterns and to have a high sensitivity. In the present study, we applied a very simple index indicating spatial preferences. Future investigations are needed to explore the predictive value of the index (51). A follow-up investigation may show whether the eye-tracking index predicts persisting deficits or whether it just reflects spatial preferences in the acute phase. The eye-tracking index of free exploration may also be a sensitive measure for tracking spontaneous recovery or progress in the therapeutic setting. Moreover, a follow-up investigation may assess the relation between the eye-tracking index and everyday-life competence which may be evaluated only in subacute or chronic phases after stroke.

## Ethics Statement

All subjects gave written informed consent in accordance with the Declaration of Helsinki. The protocol was approved by the ethics committee of Medical University Innsbruck.

## Author Contributions

MD contributed to the study concept, analysis, interpretation of data, and writing the manuscript. MS contributed to the study concept, carried out the acquisition of patients’ data, and was involved in the data analysis and the revision of the manuscript. TB performed the lesion analysis, PE assisted in eye-tracking data acquisition and analysis, CB provided clinical data and patient classification. All authors contributed to the critical revision of the manuscript and approved the final manuscript.

## Conflict of Interest Statement

The authors declare that the research was conducted in the absence of any commercial or financial relationships that could be construed as a potential conflict of interest.

## Footnotes

^1^http://www.mccauslandcenter.sc.edu/crnl/tools/cancel (Accessed: May 7, 2018).

^2^http://www.sph.sc.edu/comd/rorden/mricro.html.

^3^http://www.fil.ion.ucl.ac.uk/spm.

^4^http://www.mathworks.com.

^5^https://www.tobiipro.com/siteassets/tobii-pro/user-manuals/tobii-pro-studio-user-manual.pdf (Accessed: June 5, 2018).

^6^NE patients and NN patients did not show significant differences in fixation durations between right and left AOI, controls had shorter fixations in the left AOI in the free exploration task, but not in the search task (*p*=0.013; Wilcoxon tests).
